# Picosecond x-ray strain rosette reveals direct laser excitation of coherent transverse acoustic phonons

**DOI:** 10.1038/srep19140

**Published:** 2016-01-11

**Authors:** Sooheyong Lee, G. Jackson Williams, Maria I. Campana, Donald A. Walko, Eric C. Landahl

**Affiliations:** 1 Frontier in Extreme Physics, Korea Research Institute of Standards and Science (KRISS), Daejeon 305-600, Korea; 2 Department of Physics, DePaul University, Chicago, Illinois 60614, USA; 3 Advanced Photon Source, Argonne National Laboratory, Argonne, Illinois 60439, USA

## Abstract

Using a strain-rosette, we demonstrate the existence of transverse strain using time-resolved x-ray diffraction from multiple Bragg reflections in laser-excited bulk gallium arsenide. We find that anisotropic strain is responsible for a considerable fraction of the total lattice motion at early times before thermal equilibrium is achieved. Our measurements are described by a new model where the Poisson ratio drives transverse motion, resulting in the creation of shear waves without the need for an indirect process such as mode conversion at an interface. Using the same excitation geometry with the narrow-gap semiconductor indium antimonide, we detected coherent transverse acoustic oscillations at frequencies of several GHz.

Following the absorption of femtosecond laser radiation, bulk semiconductors respond with initial impulsive strain waves and eventual heating of the lattice. Reflections of these waves from buried interfaces are used for high-frequency ultrasonics as well as nanoscale tribology. The generation of these waves has been studied using a wide variety of pump-probe techniques, including Time-Resolved X-Ray Diffraction (TRXD)^1^,^2^. However, the interpretation of these studies has generally neglected transverse and shear strain properties, assuming a uniaxial spatial profile^3^. The uniaxial strain model is known to break down under certain situations such as when single crystals are subjected to 100 GPa shock compression^4^, in asymmetrically cut crystals with an asymmetric strain tensor^5^, when the laser is focused to small lateral spot sizes comparable to the laser penetration depth^6^, or within individual nanocrystals^7^. In particular, ultrafast all optical pump-probe methods have been employed to generate and detect transverse and quasi-transverse (QT) polarised acoustic phonons in superlattices grown on GaAs^8^,^9^,^10^,^11^. While such experiments have demonstrated detection of very high frequency QT folded phonon modes that are in the regimes of hundreds of Gigahertz, a room measurement in a bulk system has not yet been reported to our knowledge. In this work, we show for the first time that large-amplitude coherent transverse lattice displacements can be directly generated in bulk semiconductors where the dominant generation mechanism can be attributed to a universal phenomenon: the Poisson effect.

Although both longitudinal and shear strain waves are permitted by continuum elasticity theory^12^, ultrasonic measurements in many contexts are often limited to the longitudinal polarization. Both the utility and difficulty of shear wave generation is due in part to the shear modulus, which is weak compared to the Young’s modulus for many materials and interfaces. Detection of shear waves also presents challenges. Shear waves are isovolumetric, and as such can not be measured by techniques that are primarily sensitive to density modulations, including most measurements of optical properties. Therefore a common approach for both generation and detection of shear waves is to use mode conversion at non-normal acoustic interfaces^13^. This approach places restrictions on the directions and materials that may be probed, although these requirements may be relaxed in practice by coating surfaces with metal films containing a broad distribution of crystallite orientations at the interface^14^. Rather than using acoustically asymmetric interfaces, another approach is to exploit an anisotropic property in a bulk crystal^15^. For instance, shear strain waves may be optically excited and detected in zinc due to its highly anisotropic uniaxial thermal expansion^5^, and in wurzite via anisotropic piezoelectric transduction^16^. One recent application has been the use of ultrafast above-band excitation and below-band reflectometry to measure longitudinal and shear wave propagation, and thereby determine the elastic constants of the multiferroic BiFeO_3_^17^.

Atomistic models also identify both longitudinal and transverse acoustic phonon modes. Recently it has become possible to directly populate these modes in bulk semiconductors using impulsive laser excitation and subsequently observe coupling between phonon polarizations using time-resolved x-ray diffuse scattering^18^,^19^. These results seem somewhat surprising in light of the photoinduced strain model originally proposed by Thomsen^20^. That is, if the illuminated area of the pump laser beam on the sample surface is much larger than the optical penetration depth, the strain gradient along the surface normal is expected to be much steeper than along the lateral direction, so the strain may be treated one-dimensionally. These diffuse scattering results suggest revisiting the uniaxial continuum model to incorporate longitudinal-to-transverse strain coupling on a macroscopic scale (i.e., the Poisson effect). Further evidence for this coupling has come from TRXD studies using laser-produced plasma x-ray sources. A detailed lineshape analysis of the [002] reflection from LuMnO_3_ required modification of the Thomsen model to include a longitudinal-to-transverse strain tensor coupling term of 9%^21^. More recently, Schick *et al*.^22^ used a burried metallic layer to send strain following laser absorption into a ferroelectric layer grown on top. By simultaneously mapping the reciprocal space region near a single reflection from each layer, distinct transverse lattice motion could be resolved. The magnitude of this motion was consistent with a lateral contraction of the top layer’s ferroelectric mosaic blocks driven by the Poisson effect.

## Results

**Figure 1:**
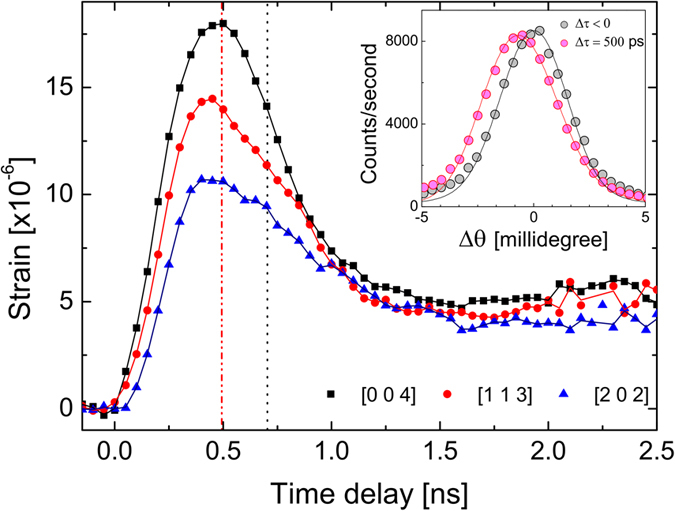
The strain Δ*d*/*d* in GaAs is calculated by taking the difference in diffraction peak centroid before and after the laser strikes the sample as a function of x-ray-to-laser delay for each reflection. The inset shows one particular set of rocking curves recorded by coincidence timing x-ray bunches, before and at *t* = 500 ps after the laser, for the [004] reflection. The diffraction data (circles) are compared to a dynamical diffraction calculation (solid lines) based on a 3D strain model incorporating electronic, acoustic, and thermal effects. For a semiconductor with a bandgap close to the laser photon energy such as GaAs, both the calculated and measured rocking curves are relatively featureless and can be represented by a centroid shift that determines an average strain.

Diffraction curves from the [004] Bragg reflection before and after the photoexcitation are compared in Fig. 1 (inset). Upon the laser excitation, the Bragg peak shifts toward smaller angles and its width broadens. These changes imply an expansion of the lattice parameter and a simultaneous inhomogeneous distribution of strain the crystal. In order to extract more quantitative information about the temporal evolution of strain, the shifts of the diffraction peaks at incremental time delays are converted to strain amplitudes via Bragg’s law, 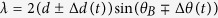 where *λ*, *d*, *θ*_*B*_, Δ*d*(*t*) and Δ*θ*(*t*) are respectively the x-ray wavelength, crystal lattice-plane spacing, Bragg angle, and changes of the lattice spacing and the Bragg angle at time delay *t*. Figure 1 shows rapid increases of mean interatomic spacing along all three directions within the first 500 ps, indicated by the first vertical dashed line in the figure. The initial expansion of the lattice is then followed by compression as the impulsive bipolar strain propagates away from the surface at the speed of sound. Notably, in the [113] and [202] x-ray reflections this acoustic propagation appears to have have a second smaller component appearing at 710 ps, which is indicated by the second vertical dashed line in the figure. This provides the first evidence that a complete model of the lattice dynamics will require two sound speeds differing by 500 ps/710 ps = 70%, which the same as the ratio of the transverse to longitudinal sound speed in GaAs. Based on the Young’s modulus of 86 GPa, a strain magnitude such as this can be achieved from an instantaneous stress on the order of megapascals, well below the plastic deformation limit. At time delays greater than 2.5 ns however, the strains along three different directions start to converge to a transient equilibrium value of 4.5 × 10^−6^.

### Picosecond x-ray strain rosette

**Figure 2:**
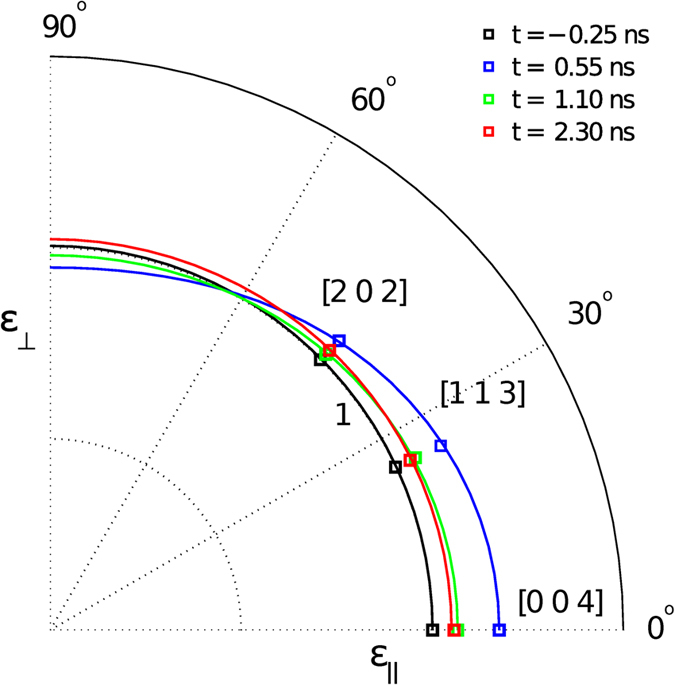
A graphical illustration for constructing the x-ray strain rosette showing the strain ellipse changing its shape as a function of time. We note that the effect of the strain is exaggerated to enhance its visibility.

To decompose the strain into its principal components, we generate a time-resolved strain rosette based on the strain amplitudes measured along three different directions with incremental time-delays of 50 ps, of which details are elaborated in the Methods section. The strains in the bulk solid averaged over the x-ray extinction depth in two dimensions are


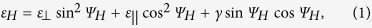



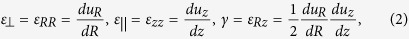


where du_*R*_, u_*z*_, are displacement components along lateral and surface directions, *ε*_*H*_ is the measured strain for the given reflection *H* at polar angle *Ψ*_*H*_ for a given time delay. The in-plane and out-of-plane components of strain are *ε*_⊥_ and *ε*_||_ respectively, while *γ* is the third principle strain component, or shear. Without making any *a priori* assumptions about the elastic models, the principal strains *ε*_⊥_, *ε*_||_, and *γ* can be obtained by simultaneous solution of Eq. (1) using experimental data. Figure 2 shows a graphical construction of the time-resolved x-ray strain rosette, in which the strain ellipse changing shape as a function of time-delays between the laser and x-ray pulses.

**Figure 3:**
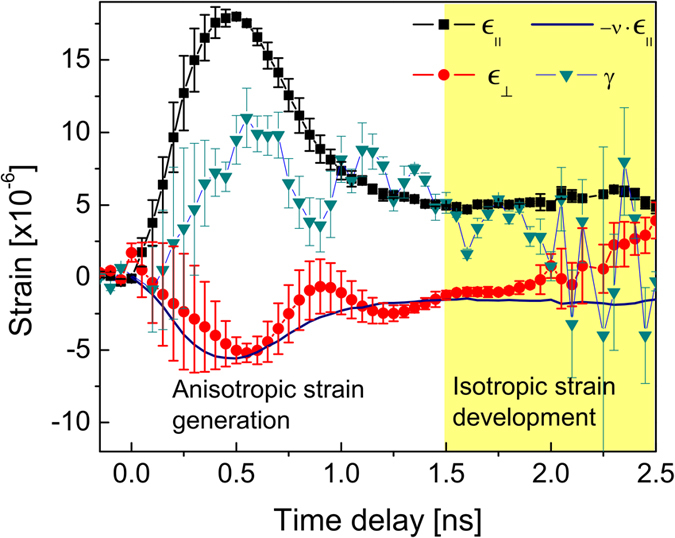
Time-resolved strain rosette decomposition for the longitudinal, transverse, and shear components in GaAs. The transverse component may be estimated at early times, when the dynamics are dominated by impulsively driven strain, by multiplying the longitudinal component by the Poisson ratio of GaAs. At later times the impulsive strain waves have left the x-ray probe depth so that the Poisson effect no longer drives the dynamics, and the crystal approaches isotropic strain. The main contributions of residual error in the decomposition are the inability of the strain rosette to handle slightly different x-ray extinction depths for different reflections combined with non-uniform strain throughout the probe depth. In particular, the difference between the longitudinal and transverse sound speeds results in different depth-dependent strains in the different directions. This mismatch is responsible for the oscillatory appearance of the shear and transverse strain components.

The results of this decomposition are shown in Fig. 3. There are several striking features apparent in the time-resolved strain rosette. First, the magnitudes of the transverse and shear strain are a significant fraction of the longitudinal strain, in contrast to the Thomsen model which would predict purely uniaxial strain with any non-longitudinal strain components equal to zero. Second, the lattice dynamics are different in different directions. Although this can also be seen by carefully inspecting the differing strain decay times in Fig. 1, the strain rosette enhances the large differences along the principal axes. Finally, the longitudinal and transverse strain components, which have opposing signs before 1.5 ns, then begin converging to the same value.

Assuming that traveling, impulsive strain dominates the lattice dynamics, we expect the coupling between longitudinal strain, *ε*_||_, and transverse strain, *ε*_⊥_, to be given by the Poisson ratio (0.31 for GaAs). In fact, at early times up to roughly 1.5 ns, it can be seen that this relation approximately holds. This corresponds to the time duration necessary for the acoustic impulsive longitudinal strain (with a spatial extent of 1 *μ*m, equal to the optical penetration depth of the laser) to propagate away from the x-ray probe depth. These results suggest a division of timescales for strain dynamics into two distinct regimes. First we refer to the earlier timescale as the “anisotropic strain regime,” where the principal longitudinal compressional wave (or “P wave”) generates motion in the transverse direction via the Poisson effect. We refer to the relatively later dynamics (after 1.5 ns) as the “isotropic strain regime” during which the longitudinal and transverse strains approach the same value and eventually uniform cooling of the crystal surface occurs by thermal diffusion into the bulk. Within the uncertainty of our measurement, beyond the time-delay of 1.5 ns, the Poisson effect no longer seems to play significant a role because the transverse strain (red circle in Fig. 3) progressively deviates away from the lateral motion that is calculated based on the Poisson ratio (solid black line). However the residuals from the shear strain persists until 2.5 ns, corresponding to the time duration necessary for a shear wave (or “S wave”) to propagate away from the x-ray probe depth at the transverse speed of sound along [001]. We note that, while it may be possible that QT modes^8^,^9^,^10^,^11^ contribute to the presence of the strains along the transverse direction, the time-scale of our measurement is considerably slower (GHz or less) as compared to those of previously reported. Quantitative agreement between our data and the Poisson ratio model indicates that the Poisson effect may be one such mechanism controlling the longitudinal-to-transverse coupling. An investigation on the combined effects of the QT mode and Poisson contributions will require femtosecond x-ray time-resolution, which is beyond the scope of our present study.

### Comparison with numerical simulation

**Figure 4:**
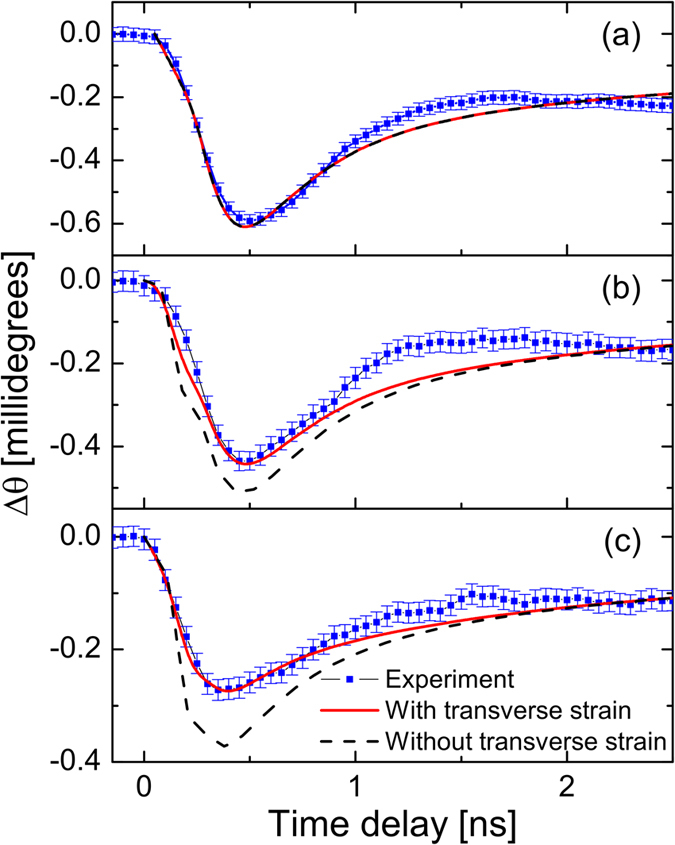
Comparison between data and simulation for the (**a**) [0 0 4], (**b**)[1 1 3] and (**c**)[2 0 2] reflections of GaAs. Symmetric reflections such as [0 0 4] which have been the emphasis of previous work do not require simulating transverse strain.

In Fig. 4, time-dependent shifts of the diffraction peaks from the experiment are compared to a strain model and dynamical diffraction calculation described in the Supplemental Materials. Quantitative agreement exists between the [004] reflection and a uniaxial strain model because the reflecting plane is parallel to the surface (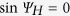), where x-ray diffraction signal is not sensitive to any motion transverse to the lattice planes. However, along the off-surface normal directions, replicating our results requires the full anisotropic strain model. This is clearest for the [202] reflection data, which is the most sensitive to transverse strain: at all time-points the anisotropic model fits better than the uniaxial strain model.

### Coherent oscillation along transverse direction

**Figure 5:**
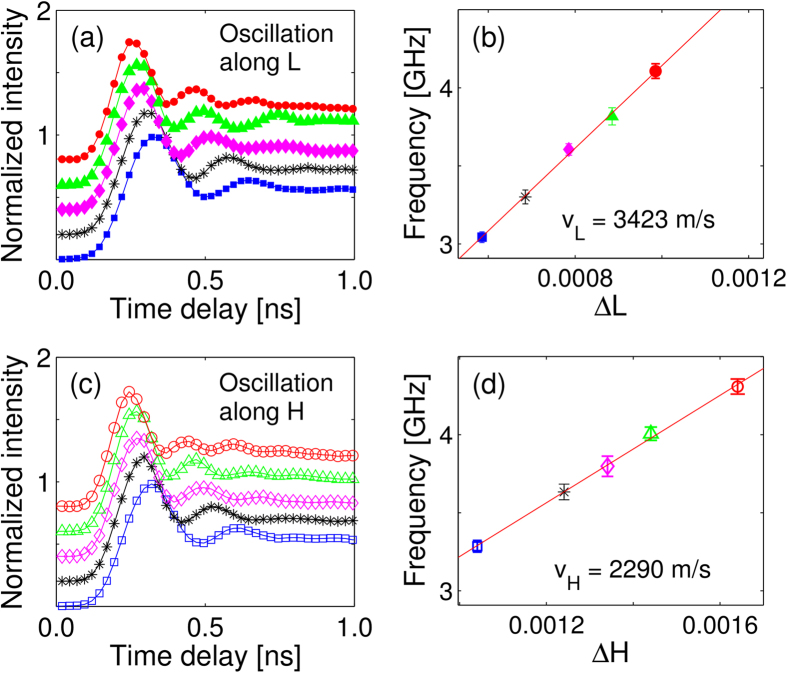
Coherent longitudinal (**a**) and transverse (**c**) lattice oscillations observed in InSb by TRXD near the[2 0 2] reflection. The diffracted x-ray count rates before and after the laser strikes the sample are deadtime corrected and subtracted, and normalized by the maximum relative signal. Increasing angles away from the[2 0 2] peak in either the H (transverse) or L (longitudinal) direction (i.e., [2 + Δ*H* 0 2] or [2 0 2 + Δ*L*]) are represented by increasing vertical offsets for clarity. The value of each offset Δ*L* and Δ*H* in (**a**,**c**) is given by the matching symbols in (**b**,**d**), respectively. Peak fitting the first two oscillations of each delay scan in (**a**,**c**) determines a phonon frequency corresponding to a point on the dispersion curves in (**b**,**d**).

In bulk materials, the strain-rosette approach is most readily applied where the laser pump and x-ray probe penetration depths happen to be matched, such as the 800 nm wavelength laser excitation of GaAs discussed above where both distances are on the order of 1 *μ*m. (X-ray extinction and absorption depths for all GaAs reflections used are tabulated in the Supplemental Materials.) Although the strain rosette provides quantitative measurement of the 3D strain profile near the surface, we can only infer the propagation of the shear wave from this data. Direct observation of wave behavior requires the x-rays to probe several oscillatory wavelengths. We demonstrated this by using a single crystal of (001) cut indium antimonide (InSb), a narrow-gap semiconductor with an optical penetration depth (91 nm) that is approximately an order of magnitude shorter than the x-ray extinction depth at 8 keV. An incident laser fluence of ~1 mJ/cm^2^ was used to excite lattice motion in all scans. We chose to measure near the [202] reflection where diffraction intensities are equally determined by the H (transverse) and L (longitudinal) lattice displacement. At fixed angular offsets in the longitudinal direction, denoted as a shift of Δ*L* in reciprocal lattice units (i.e., [2 0 2 + Δ*L*]), we observed oscillations in the normalized diffracted intensity as the time delay was scanned (Fig. 5a). The oscillatory signals occur because of presence of coherent phonons^2^,^23^. Generation of an impulsive stress excites broad bands of acoustics modes that are in-phase upon femtosecond laser excitation. The frequency *f* of these oscillations increases with increasing angle and yields the dispersion relationship shown in in Fig. 5b from which the speed of sound 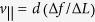 is obtained.

For the longitudinal angular offsets in L, the measured slope of 3423 ± 93 m/s agrees with the longitudinal speed of sound along the [001] direction for InSb, *v*_||_ = 3400 m/s. Our methods and results for this measurement are the same as those previously reported for InSb^24^. Time scans at fixed angular offsets in the transverse direction (Fig. 5c) also showed oscillations in the diffracted intensity, but this time with a different dispersion relationship 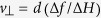 (Fig. 5d). In this case the measured slope of 2290 ± 55 m/s agrees with the transverse speed of sound along the [001] direction for InSb, *v*_⊥_ = 2290 m/s. The observed damping time for these coherent transverse and longitudinal acoustic phonons corresponds to their transit time out of the x-ray probe depth and limits the lowest frequencies we can measure. The highest frequency phonons observed (~6 GHz) correspond to the 100 ps temporal resolution of our synchrotron x-ray probe. The coherent transverse acoustic speed does not agree with any other phonon mode or polarization near the Brillouin Zone center. The origin of the transverse oscillation may trace back to the initial excitation of the lattice. Upon femtosecond laser excitation, various modes including optical phonons as well as terahertz acoustic phonons at Brillouin zone edges can be excited, which later couple to lower frequency phonon-modes. Nevertheless, current time-resolution of our measurement at APS is limited to probing GHz lattice vibrations. And thus, understanding the origins of the transverse coherent phonons remains a topic for future study.

## Discussion

Large transverse motions generated via the Poisson effect and the resulting shear acoustic waves are found even in the uniaxial spatial profile^3^ and at relatively low excitation intensities. Shear and longitudinal waves can be distinguished both temporally and directionally using TRXD. In future studies, it will be very beneficial to investigate how the relative sizes of the pump beam respect to the probe beam effect strain generation and propagation dynamics for more systematic understanding of the phenomena. Future work will also develop 3D picosecond ultrasonics by increasing the pump-probe delay to match acoustic reflection times from buried interfaces. Applications include the nondestructive evaluation of patterned nanoscale buried interfaces, which should exhibit distinctive acoustic signatures perpendicular to their growth direction. At higher laser intensities we would expect the significant amount of transverse kinetic energy present to enhance both nonlinear and anisotropic properties such as the deformation potential tensor. For instance, it has been postulated that transverse motion is required for the nonthermal phase transition in GaAs from a zincblende to simple cubic structure following intense laser irradiation^25^. Finally, the extension of these methods to sub-picosecond x-ray durations should allow the study of non-equilibrium continuum mechanics at early times and atomic resolution before the Poisson effect has permitted a material to respond to applied stress. Ultimately, more advanced light source facilities such as a hard x-ray FEL^26^ may be required to study such high-speed mechanism.

## Methods

### Construction of time-resolved x-ray strain rosette

Anisotropic strains in continuum mechanics may be measured using a strain rosette, or a set of noncolinear strain gauges that measure the same elastic volume^27^. To construct a strain rosette at atomic scales and picosecond time resolution, we measured time-resolved x-ray diffraction rocking curves (or *θ* scans) from three different lattice planes of a single-crystal (001) cut GaAs wafer following excitation by an 800-nm wavelength, 50-fs amplified Ti:sapphire laser operating at a 5-kHz repetition rate. Synchrotron TRXD was performed at Beamline 7ID of the Advanced Photon Source^28^ where a water-cooled double-crystal diamond [111] monochromator combined with horizontal-plane focusing and vertical slits provided a collimated, 50-micron square 8-keV x-ray beam on the semiconductor sample. Before and after the diffraction measurements, knife-edge scans of both laser and x-ray beams at the sample position verified that the laser uniformly overfilled the x-ray spot with a 5-mm FWHM smooth spatial profile. With the use of four-circle diffraction geometry, the sample was oriented in the x-ray beam such that the [004], [113], and [202] reflections could be measured near their intrinsic angular resolution. X-ray intensity was measured both before the sample from an upstream scattering polyimide foil for normalization as well as 1 m after the sample on the diffractometer arm, which used collimating slits to reduce background scatter. Absorbing filters were also placed on the detector arm to reduce the x-ray intensity so that an extended dead-time model^29^ could be used for dead-time correction and error estimation. Both x-ray detectors were single photon counting avalanche photodiodes capable of timing discrimination between successive x-ray pulses (separated by 153 ns). The amplified laser pulses were synchronized to the x-ray beam such that arbitrary laser–x-ray delay times, *t*, could be adjusted electronically with <1 ps precision and directed onto the sample surface colinearly with the x-ray beam. Measuring a strain rosette requires that the same conditions, or total absorbed fluence (chosen to be *F* = 0.42 mJ/cm^2^), be maintained for each condition. We accomplished this by keeping the laser focusing optics and incident beam path the same for each reflection, but controlling the total laser intensity by adjusting a half wave plate followed by a polarizing beam splitter. Two corrections had to be made for each reflection: one for the differing Fresnel reflectivity which was measured in place using an optical power meter, and one for different laser beam size footprints on the sample. For this correction, the real-space incident angles of each reflection were used to determine the projection of the laser beam across the sample and thereby determine the proper incident laser intensity.

### Simulation

See our Supplemental Materials

## Additional Information

**How to cite this article**: Lee, S. *et al*. Picosecond x-ray strain rosette reveals direct laser excitation of coherent transverse acoustic phonons. *Sci. Rep*. **6**, 19140; doi: 10.1038/srep19140 (2016).

## Supplementary Material

Supplementary Information
