# Supplementary material for: Picosecond x-ray strain rosette reveals direct laser excitation of coherent transverse acoustic phonons
Source: Sci Rep. 2016 Jan 11;6:19140. doi: 10.1038/srep19140 (PMC4707471; doi:10.1038/srep19140)
Supplement: Supplementary Information [file srep19140-s1.pdf]

Supplementary Materials for "Picosecond X-ray Strain Rosette Reveals Direct Laser Excitation of Coherent Transverse Acoustic Phonons"

Sooheyong Lee,[1] G. Jackson Williams,[2,*] Maria I. Campana,[2] Donald A. Walko,[3] and Eric C. Landahl[2]

[1]*Korea Research Institute of Standards and Science (KRISS), Daejeon 305-600, Korea*

[2]*Department of Physics, DePaul University, Chicago, Illinois 60614, USA*

[3]*Advanced Photon Source, Argonne National Laboratory, Argonne, Illinois 60439, USA*

(Dated: October 7, 2015)

INTRODUCTION

We present our numerical scheme for simulating time-resolved x-ray diffraction in GaAs with laser-induced strain generation and propagation perpendicular (symmetric reflection) as well as non-perpendicular (non-symmetric reflection) to the surface orientation. We employ a model assuming electron-hole plasma and impulsive strain generation upon laser excitation followed by relaxation dynamics such as Auger recombination and free-carrier and thermal diffusion. The Poisson effect is used to couple longitudinal to uniaxial strain. The effect of the transient strain field on the x-ray diffraction curves for three non-collinear reflections [004], [113], and [202] are calculated using the method proposed by Wie et al. [1].

UNIAXIAL STRAIN MODEL

In order to simulate the strain dynamics, we begin with a 1D carrier-driven model for longitudinal strain in semiconductors consisting of impulsive strain propagation and lattice relaxation via carrier diffusion and recombination described previously [2]. When a semiconductor crystal is illuminated by a laser beam with photon energy greater than the electronic energy bandgap, free carriers are generated within the optical penetration depth. With the availability of femtosecond laser pulses, an electron-hole plasma density of 10^{14} to 10^{19} cm^{-3} can be achieved, which is sufficient to induce impulsive strains due to the deformation potential and free-carrier diffusion into the bulk crystal followed by relatively slow thermal diffusion.

In our strain model, two assumptions are made: (i) upon the femtosecond laser excitation on the crystal, the energy relaxation from the laser photons to creating the electron-hole plasma takes place instantaneously, and (ii) the electron-hole pairs relay their energy immediately to the lattice.

These are reasonable approximations because these processes occur on a sub-picosecond time scale, which is much shorter than the time-resolution of a synchrotron x-ray pulse (90 ps FWHM). A portion of the laser photon energy, E_p, is used to promote electrons from the valence band to the conduction band while the excess energy $E_p - E_g$ is transferred to the lattice as heat, where E_g is the electronic energy band gap. At a relatively low excitation level below the carrier saturation, the laser energy is deposited on the crystal surface within the 1/e absorption depth, ζ. The initial free carrier density and temperature profiles are given by

$$\begin{aligned} n(z,t=0) &= \frac{F}{E_p \zeta} e^{-z/\zeta} \\ T(z,t=0) &= \frac{E_p - E_g}{C_l} n(z,t=0) \end{aligned} \tag{1}$$

where $n(z,t)$ is the free carrier density, $T(z,t)$ is the lattice temperature and C_l is the lattice heat capacity per unit volume. Using an absorbed fluence of $F = 0.42$ mJ/cm^2 and $E_p = 1.55$ eV, we expect to have a carrier density of 2×10^{19} cm^{-3} within the optical penetration depth.

Transient removal of the carrier population takes place via non-radiative Auger decay, radiative recombination and ambipolar diffusion which are characterized by the constants A, B and D_p respectively. This results in lattice heating and eventual thermal diffusion, D_t, characterized by

$$\begin{aligned} \frac{\partial n}{\partial t} &= D_p \frac{\partial^2 n}{\partial z^2} - An^3 - Bn^2 \\ \frac{\partial T}{\partial t} &= D_t \frac{\partial^2 T}{\partial z^2} + An^3 \frac{E_g}{C_l}. \end{aligned} \tag{2}$$

The electronic strain is generated both from the free carriers via the deformation potential [3], α_p and temperature via the thermal expansion coefficient, α_t,

$$\epsilon_e(z,t) = \alpha_p n(z,t) + \alpha_t T(z,t). \tag{3}$$

Rapid expansion of the lattice near the surface launches two counter propagating acoustic pulses along the surface normal direction. Consequently, we expect three longitudinal strain components consisting of the decaying electronic strain and two resulting traveling strain components ϵ_+ and ϵ_-, where a π phase shift exists upon reflection from the surface of the crystal, and the longitudinal strain is given by

$$\epsilon_{||}(z,t) = \epsilon_e(z,t) + \epsilon_+(z,t) + \epsilon_-(z,t). \tag{4}$$

3D STRAIN MODEL

Extending this model to describe transverse strain requires three assumptions. First, we presume that the electronic strain is isotropic, which is consistent with a picture of the deformation

potential exerting uniform pressure in all directions in response to the sudden generation of free charge carriers. Second, we recognize that the 1D initial conditions also permit a shear wave along the surface normal direction [4]. Third, we propose that this transverse strain is driven by the Poisson ratio ν, which is an elastic response to the longitudinal lattice compression such that

$$\epsilon_{\perp}(z,t) = \epsilon_e(z,t) - \nu\left[\epsilon_+(z,t) + \epsilon_-(z,t)\right]. \tag{5}$$

The material parameters listed in Table I were used to calculate the transient response of the lattice. First, Eq. 2 is solved numerically to give the electron density and thermal profiles at each time point. This is inserted into Eq. 3 to calculate the total generated strain, which is then allowed to propagate according to the elastic wave equation.

X-RAY DIFFRACTION CURVE CALCULATION

The strain rosette analysis shown in Fig. 2 of the main text was performed using the kinematical approximation where the strain is spatially averaged over the x-ray probe depth. To calculate the transient evolution of x-ray diffraction curves, the strain is incorporated into dynamical diffraction theory that takes into account both the time and depth dependence of our strain model. Since the [113] and [202] reflections have orthogonal components to the surface plane, the effect of transverse strain is incorporated in our TRXD simulation, as well as the asymmetry angle, ψ_A. The differential Bragg angle for cubic crystals of arbitrary orientation,

$$\begin{aligned}\Delta\theta(z,t) = &-\left[\epsilon_{\perp}(z,t)\sin^2\psi_H + \epsilon_{\|}(z,t)\cos^2\psi_H\right]\tan\theta_B \\ &+\left[\epsilon_{\perp}(z,t) - \epsilon_{\|}(z,t)\right]\sin\psi_A\cos\psi_A,\end{aligned} \tag{6}$$

is used to calculate the time-resolved diffraction curves.

For each reflection, the position and lineshape of the x-ray diffraction curves are calculated numerically using the formulation derived by Wie et al.[1, 5], in which depth-dependent strain fields are integrated into the solution of the Takagi-Taupin equations. In previous time-resolved x-ray simulations, where mostly symmetric reflection geometries have been investigated, the transverse strain term has been left out for simplicity. In our simulations, numerical depth step size is kept sufficiently thin such that the maximum strain change within a single layer does not exceed 3×10^{-8} per time step. Table II shows the list of parameters used for the x-ray diffraction calculations and Fig. 1 shows the results of the simulations for different diffraction planes. Peak shifts for each reflection were determined by applying an asymmetric Gaussian fit to the x-ray diffraction curves.

* Current Affiliation: Lawrence Livermore National Laboratory, Livermore, California 94550-9234,USA

[1] C. R. Wie, T. A. Tombrello, and T. Vreeland, Jr., Journal of Applied Physics **59**, 3743 (1986).

[2] M. F. DeCamp, D. A. Reis, A. Cavalieri, P. H. Bucksbaum, R. Clarke, R. Merlin, E. M. Dufresne, D. A. Arms, A. M. Lindenberg, A. G. MacPhee, Z. Chang, B. Lings, J. S. Wark, and S. Fahy, Phys. Rev. Lett. **91**, 165502 (2003).

[3] P. Basu, *Theory of Optical Processes in Semiconductors: Bulk and Microstructures*, Vol. 4 (Oxford University Press, USA, 1998).

[4] L. D. Landau and E. M. Lifshitz, *Theory of Elasticity*, Vol. 7 (1986).

[5] V. S. Speriosu and T. Vreeland Jr., Journal of Applied Physics **56**, 1591 (1984).

[6] U. Strauss, W. W. Ruhle, and K. Kohler, Applied Physics Letters **62**, 55 (1993).

[7] B. A. Ruzicka, L. K. Werake, H. Samassekou, and H. Zhao, Applied Physics Letters **97**, 262119 (2010).

TABLES AND FIGURES

Lattice parameter	5.65325 Å
Energy band gap	1.42 eV
Laser absorption depth, 1/e	1 μm
Auger recombination rate	4×10^{-38} cm^6/ns [6]
Radiative recombination rate	1.7×10^{-19} cm^3/ns [6]
Deformation potential coefficient	5×10^{-24} cm^3 [3]
Ambipolar diffusion coefficient	1 μm^2/ns [7]
Thermal diffusivity	0.03 μm^2/ns
Crystalline heat capacity	1.13×10^{19} eV/cm^3 K
Longitudinal sound speed	4.73 cm/s
Transverse sound speed	3.35 cm/s
Poisson ratio	0.31
Absorbed laser fluence	0.42 mJ/cm^2

TABLE I. Table I. GaAs material parameters used for the simulation.

Reflection $[h\ k\ l]$	[0 0 4]	[1 1 3]	[2 0 2]
θ_B [deg]	33.2496	27.0402	22.8113
ψ_H [deg]	0	25.2394	45
ψ_A [deg]	0	4.19	5.13
Absorption factor [1/cm]	348.5		
Absorption depth [μm]	24.0	25.5	26.5
Extinction depth [μm]	1.5	1.6	0.9
F_0	247.1 + 7.2i		
F_{hkl}	150.4 + 7.0i	76.2 + 89.7i	179.0 + 7.1i

TABLE II. Table II. GaAs parameters that were used for the diffraction calculations.

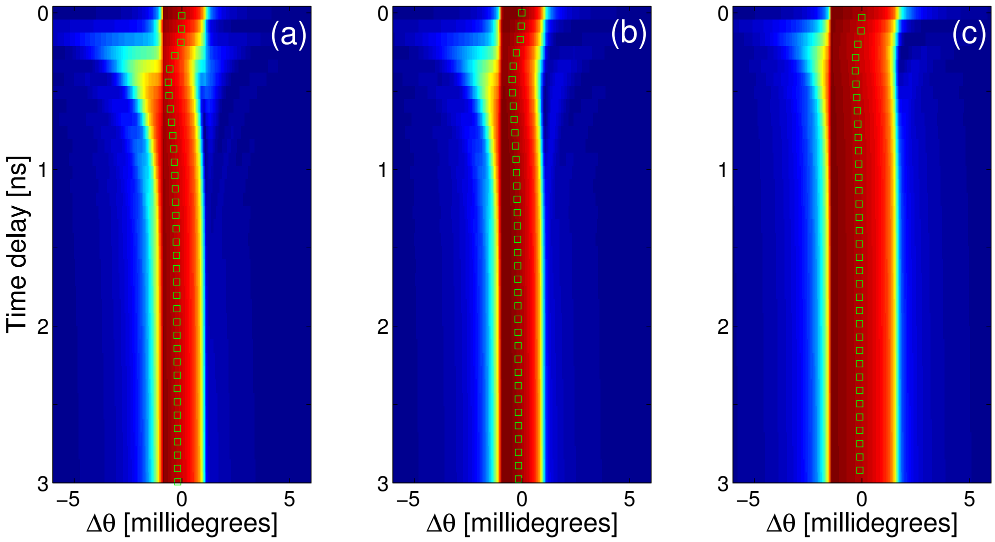

FIG. 1. Simulated time-resolved diffraction curves of optically excited GaAs, for Bragg reflections (a) [004], (b) [113], and (c) [202]. Square symbols mark the fitted center of the peak at each time point, for the parameters listed in Table I and II.
